# A Survey on One Health Approach in Colombia and Some Latin American Countries: From a Fragmented Health Organization to an Integrated Health Response to Global Challenges

**DOI:** 10.3389/fpubh.2021.649240

**Published:** 2021-10-25

**Authors:** Natalia Margarita Cediel Becerra, Ana María Olaya Medellin, Laura Tomassone, Francesco Chiesa, Daniele De Meneghi

**Affiliations:** ^1^Epidemiology and Public Health Research Group, Veterinary Medicine Program, School of Agricultural Sciences, Universidad de La Salle, Bogotá, Colombia; ^2^Veterinary Medicine Program, School of Agricultural Sciences, Universidad de La Salle, Bogotá, Colombia; ^3^Department of Veterinary Science, University of Turin, Grugliasco-Turin, Italy

**Keywords:** intersectoral collaboration, Latin America countries, one health, questionnaire survey, perception, barriers, Colombia

## Abstract

The “One Health” (OH) approach has been recognized by world health authorities such as FAO/OIE/WHO, advocating for effective, multi-sectoral, and transdisciplinary collaboration. However, there is a lack of published evidence of the awareness of the OH concept in Colombia and other countries in the Latin American Region. In order to explore existing collaboration amongst the animal health, human-public health, environmental health sectors, and to describe the perception, knowledge, and barriers on OH in Colombia and other countries of Latin America, an online questionnaire-based survey was distributed among key professionals representing the three OH pillars (August 2018–August 2020). Overall, 76 key respondents from 13 countries (Colombia, México, Chile, Brazil, Argentina, Bolivia, Costa Rica, Ecuador, Perú, Guatemala, Nicaragua Uruguay, and Venezuela) completed the questionnaire. Respondents worked in institutions of animal (59%), public (20%), human (7%), and environmental health (7%); they mainly belonged to higher academic institutions (59%), followed by ministries (11%), and research organizations (9%). Most participants (92%) were familiar with the OH term and 68% were aware of the formal cooperation among sectors in their countries, mostly on zoonoses; in 46% of the cases, such connections were established in the last 5 years. The main reported limiting factors to intersectorality were the lack of commitment of policy-makers, resources, and budget for OH (38%) and the “siloed approach” of sectors and disciplines (34%). Respondents ranked a median score of 3.0 (1–5 scoring) in how good OH activities are implemented in their countries, and a median score of 2.0 in the citizen awareness on OH as regards their countries. The most important OH issues were identified in vector-borne diseases, rabies, wrong and/or improper use of antimicrobials, emerging viral diseases, food-borne diseases, neglected parasitic diseases, deforestation, and ecosystem fragmentation. Although there is a high-perceived importance on conjoint cooperation, OH implementation, and operationalization remain weak, and the environmental component is not well-integrated. We consider that integration and implementation of the OH Approach can support countries to improve their health policies and health governance as well as to advocate the social, economic, and environmental sustainability of the Region.

## Introduction

During the last 15 years, there has been an increased focus on the human-animal-ecosystem interface. Pathogens continue to evolve and adapt to new hosts and environments, threatening human and animal health systems. Highly pathogenic avian influenza and some other infectious diseases, COVID-19 as latest example, have created an opportunity toward a One Health (OH) approach that incorporates a collaborative, cross-sectoral, multidisciplinary mode of addressing these threats, and reducing health risks (1). Consequently, OH underwent a revival from the academy, the government, and international organizations (2).

One Health has been defined by the WHO as “an approach to designing and implementing programs, policies, legislation, and research in which multiple sectors communicate and work together to achieve better public health outcomes” (3). This definition confirms the importance of the animal-human-environment interface and how vital it is to ensure the adoption of a OH approach in public health legislation in all countries. The roots of this paradigm lie in the fertile grounds of comparative pathology, driven by the remarkable efforts, perspectives, and writings of William Osler, Calvin Schwabe, Rudolf Virchow, and many others (4). The OH approach, therefore, involves combined assessment of health risks across the three domains of humans, animals, and the environment, and it involves design and implementation of intervention and prevention strategies that address all three sectors with the goal of producing integrated knowledge (5). The OH collaboration has the potential to benefit many sectors, having among its advantages: more efficient and effective surveillance programs, better development of laboratory capacity, improved targeting efficient outbreak prediction, implementation of common disease control strategies, identifying integrated research activities across sectors (human, animal, environmental) (6).

Despite an intuitive appreciation that complex health problems need to be tackled through an integration of the interdisciplinary and transdisciplinary approaches that define OH, there is still need to generate quantitative and qualitative evidence to clearly demonstrate these benefits and its added value (7, 8). Collaborative approaches in health are promising; nevertheless, several authors point at persistent challenges for designing and implementing OH initiatives. The degree and quality of collaboration amongst various health disciplines and institutions varies substantially (9). Integrated approaches to health are challenging because they require complex systems of communication and collaboration that are difficult to delimit (9). Rüegg et al. (9), reflecting on the concrete challenges for OH implementation, described real case studies of application of OH in different countries. The most important topics to consider were related to the social dimensions and power dynamics among professional participants that affect OH implementation, the importance of local and national levels for the successful realization of OH, how the social-ecological systems and resilience theory contribute to the OH approach. However, the national borders are challenging for the sharing of epidemiologic data and systems thinking is challenging for many natural scientists (10).

In Latin America, a One Health International Network (OHLAIC) was started as an international cooperation initiative with representatives and leaders from over 20 countries. It was created in December 2017 through virtual communications between the One Health Commission (OHC), OH Platform (in connection of OH Day) and five OH representatives from Chile, Brazil, Perú, and Colombia as the co-founders. This OHLAIC Network met in person with further OH experts of representative countries in Monteria, Colombia in 2018 and 2019 (11, 12). This network goal is addressing urgent health problems in Latin America without any competitiveness between areas (https://ohlaic.org/es/) and representing English, Spanish, French, and Portuguese speaking countries. Specifically, in Colombia, the OH concept has been deepened by the Academia since the creation of SPVet network, created following a recommendation made at the First Meeting of Veterinary Public Health, held in Bogotá in 2003, under the auspices of the Representation of PAHO/WHO. During this meeting, an important dialogue was carried out related to food hygiene, prevention of zoonoses, weak public perception of the role of veterinarians in the health of society, low importance of veterinary public health in higher education, and limitation of guidelines for professional practices and the consequent fragmentation of the agricultural sector in the decision-making regarding the health system and the development of the country. The objectives of the SPVet network were: maintaining a continuous and timely flow of information on veterinary public health topics, strengthening ties of cooperation and support among specialists, create a space for discussion and consultation on topics of national interest as international, and contribute to the strengthening of undergraduate and graduate academic activities in veterinary public health with the participation in the Sapuvetnet project, an international network aimed to promote and harmonize teaching and research on Veterinary Public Health across Latin America and Europe. This international project contributed to develop and share innovative undergraduate teaching material on the importance of intersectoral collaboration and multidisciplinary cooperation to face the most important global challenges (13, 14).

There are an increasing number of researchers from universities and government agencies across many countries of the Latin American region, with different expertise and disciplinary backgrounds that may facilitate a more comprehensive perspective at the human-animal-environment interface. However, there is a gap in knowledge on the state of OH approach in Latin American countries it seems there is a lack of direction on the implementation of OH initiatives among stakeholders, despite the World Bank published guidance on how to operationalize OH (15).

In order to explore existing collaboration amongst the animal health, human-public health and environmental health sectors, and to describe the perception and knowledge on OH in Colombia and other countries in Latin America, a questionnaire-based survey was circulated amongst main stakeholders.

## Materials and Methods

Our questionnaire was derived from a similar questionnaire used to carry out a qualitative survey on OH perception and experiences in Europe and neighboring areas (16). The questionnaire aimed to assess the perception and experiences from key stakeholders on OH. This form was developed in Google Forms (https://docs.google.com/forms/) and distributed by email within the networks to which one of the authors belongs (NC): Sapuvet and the “One Health Latin American and Iberoamerica and the Caribbean network” (OHLAIC). The potential participants were reminded about compiling the questionnaire every 3 months via mail. Likewise, a personal invitation to participate in the study was sent to the major representatives of public health authorities and animal health authorities in Colombia, Mexico, and Perú through the snowball sampling technique (17). This is a recruitment technique in which research participants are asked to assist researchers in identifying other potential subjects. Likewise, several attempts were made to contact and invite the OIE Latin America and PAHO/WHO representatives to join the study.

The survey was organized in six sections: 1. *general information*; 2. *about “One Health”*; 3. *zoonotic diseases, environmental health and AMR: examples of “burning” OH issues/initiatives*; 4. *aspects limiting interdisciplinarity and intersectionality in OH*; 5. *conclusions*; 6. *end of questionnaire* (including comments, remarks and/or suggestions). The survey consisted of 27 questions, 21 closed-ended questions and 6 open-ended questions. An informed consent form was shown at the beginning of the questionnaire to warn the participants that the questionnaire was anonymous and that, by completing and submitting it, they voluntarily agreed to participate. In this way, an implicit confidentiality agreement was made with participants. Correspondingly, as our questionnaire is the same used in the survey on OH perception and experiences in Europe and neighboring areas (16), the ethical approval was granted by the Clinical Research and Ethical Review Board at the Royal Veterinary College, grant holder of COST Action TD1404 NEOH (ref. prot. n. URN 2016 1554).

The corresponding author contacted institutions and key actors and networks involved in OH in Latin America countries. Key respondents were meant to represent the three components of OH (animal, human/public, and environmental health) in each of the 21 countries, belonging to different institutions. Public institutions/ministries were represented by respondents working in the agricultural or health Ministry, veterinary services, or environmental services. We understand public health as the science of protecting and improving the health of people and their communities; public health professionals worked in areas related to the Ministry of Health (MoH), independently their college degree. Human health was defined by a state of complete physical, psychological, and social well-being; professionals working in these areas were physicians (medical doctors). Academia/research personnel (i.e., professors and researchers of the universities and national research centers), representatives of the private sector (i.e., members of the national boards/colleges of veterinarians, advisers, people belonging to the economic field selling goods or veterinary products, etc.), NGOs, associations, and scientific societies involved in OH initiatives and activities were also asked to answer the survey.

The targeted number of respondents was at least 126 (six respondents representing human, animal, and environmental health, two respondents of each component from the 21 countries of Latin America where the survey was sent). The questionnaire was accessible for 24 months (August 2018–August 2020). After the questionnaire was closed, the data collected were downloaded. Answers were checked for consistency, cleaned, and coded for analysis.

In section 3 of the questionnaire, participants were asked to select zoonotic diseases that are controlled and monitored by the MoH and/or agriculture in their respective country; the list of zoonoses has been taken from the Pan-American Health Organization (https://www.paho.org/es/temas/zoonosis).

In section 4, participants were asked to score, based on an absolute category rating, the level and opportunities for OH collaborations in their countries, choosing among: “poor*,”* “fair,” “good,” “excellent,” and “n/a”: not applicable. We present these scores in percentages at different institutional and/or professional levels.

In section 5, respondents were asked to evaluate the implementation of the OH approach by the professionals scoring from 1 (poor) to 5 (excellent); to describe formal initiatives to establish intersectoral collaboration; to give their “top 3” environmental, animal health, or public health problems in the last 5 years in their country; to name three institutions responsible for OH in their country; and finally, the level of knowledge about OH of the country inhabitants scoring from 1 (poor) to 4 (excellent). The scoring and type of scale made it possible to transfer the results from a qualitative approach to a quantitative one by giving a score to each answer (18, 19). We present the results using box-plots, to illustrate the median score (plus IQR and min/max) attributed by respondents.

Qualitative data (open questions) were analyzed using content analysis method, thus categorizing, coding, and then identifying different themes and the relationships between them. As regards the question related to One Health definition, we used the Tripartite Zoonotic Guide definition of OH (20) to identify if the answers aligned with the three health components and key terms such as: collaboration, intersectoral, multi-disciplinarity, and better design of health policies. Answers to the question asking for examples of OH initiatives were categorized using the main topics as follows: (i) zoonoses and subclassification of zoonoses (vector borne, food borne), (ii) AMR and themes related to food hygiene, (iii) animal welfare, (iv) answers that did not declare any specific topic of OH approach (e.g., belonging in a OH network or teaching the concept at some level).

Data was organized in Excel (v19) and graphics were created with Excel, Word, Displayer (online) https://www.displayr.com/, and Sankey Flow Show (online) https://www.sankeyflowshow.com/. Descriptive statistics of answers and scores was carried out. We analyzed and presented data in two ways: 1. answers from all participating countries, including Colombia, and 2. answers from all participating countries, excluding Colombia. We included the survey (Spanish version) in Supplementary Material. Also, we are available to provide raw data if requested.

## Results

### General Information

Overall, 76 respondents from 13 countries answered the questionnaire, with at least one respondent per country.

**Figure 1:**
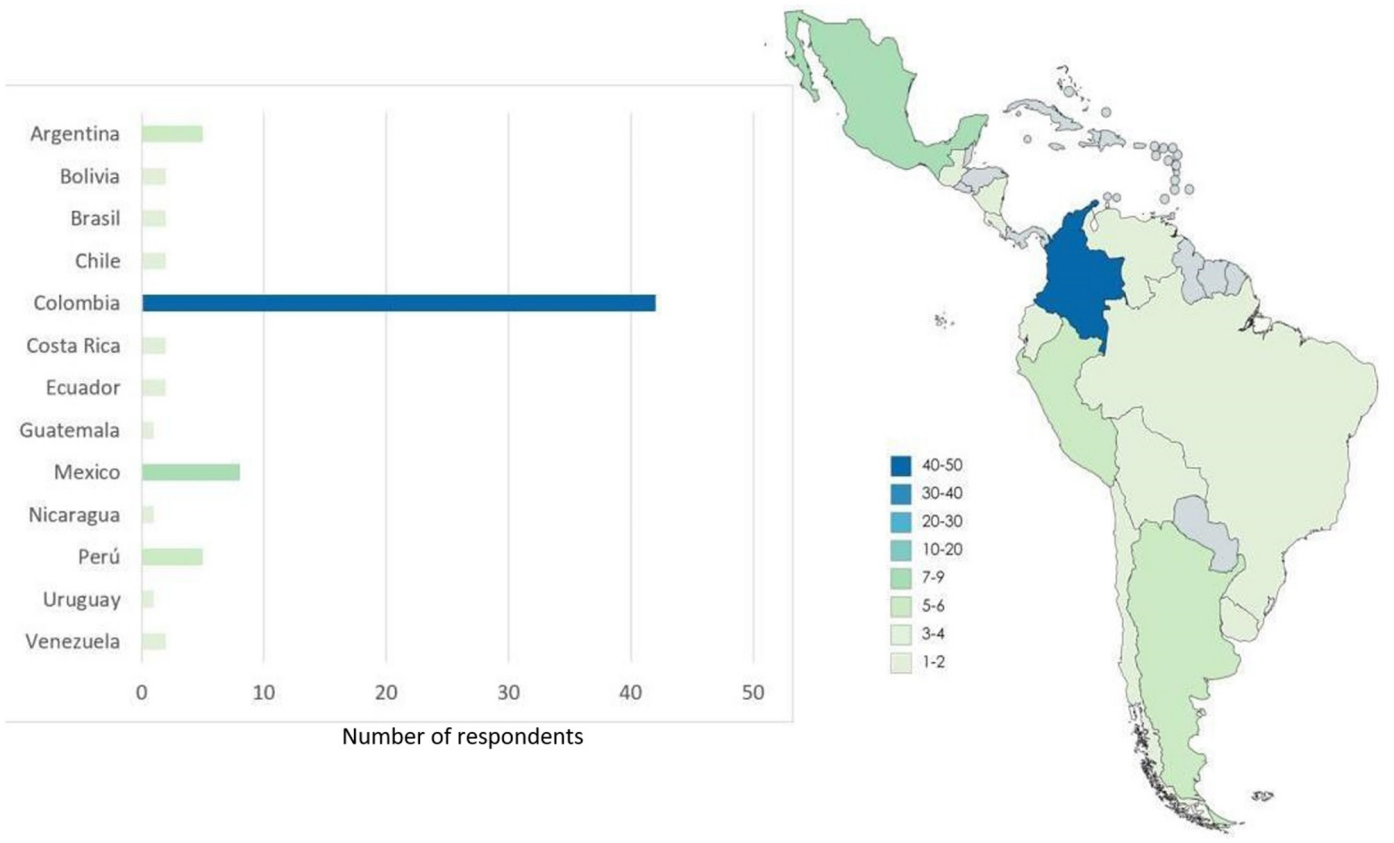
Map illustrating the number of respondents to the questionnaire per country, and bar chart with the number of questionnaires answered. Colors grade from light green (low number of questionnaires answered) to dark blue (high number).

Few countries (Colombia, Mexico, Perú, and Argentina) reached the targeted number of questionnaires answered (3), other countries reached two questionnaires answered (Ecuador, Costa Rica, Chile, Brazil, and Bolivia), and the other ones sent one answer only. Colombia had 42 answers representing 55% of the total (Figure 1). In most of the sections of the survey, the results were very similar among analysis with all countries included and without Colombia; we presented the differences when appeared.

Considering the responses from all countries (*n* = 76), almost half of the respondents had a professional degree in animal health or animal husbandry (*n* = 37, 49%), followed by public health (*n* = 31, 40%). Only two respondents stated that they had a professional degree in human health (*n* = 2, 3%). One respondent had professional training/education studies in environmental sciences (1%), one in education (1%), one in economics (1%), one in commerce (1%).

The majority of respondents worked at higher education institutions/universities (*n* = 44, 58%) and governmental institutions/ministries (*n* = 8, 11%); others in research centers (*n* = 5, 7%). Those working in NGOs were 5, 7%; without Colombia (*n* = 5; 24%) and the private sector (*n* = 2, 3%; without Colombia *n* = 2, 9%). Five respondents did not provide details.

**Figure 2:**
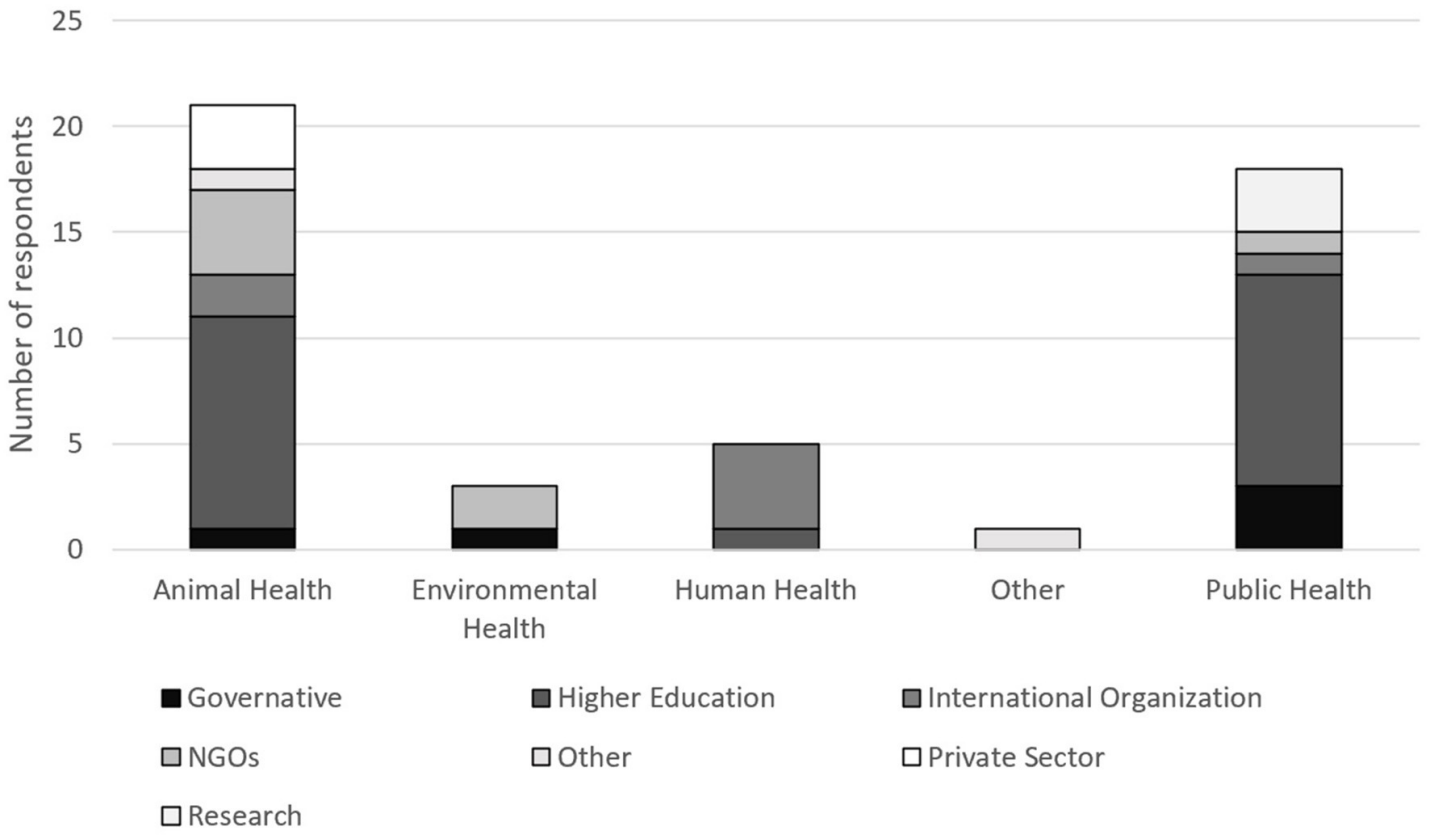
Typology and discipline of institutions by which respondents were employed.

The majority of respondents belonged to 45 institutions working in animal health (59%), followed by 15 in public health (20%), and only 7% in human health (*n* = 5) and 7% in environmental health (*n* = 5) (Figure 2). Veterinary public health respondents worked in the public health sector.

Most of the respondents stated to be professors (*n* = 26; 34%), heads/directors (*n* = 17; 22%), and researchers (*n* = 17; 22%). In less proportion, only 7% of respondents were consultants and others work as vet clinicians (7%). The students and retired compound (6%) of participants.

### About One Health

Considering all countries, 70 respondents (92%; without Colombia *n* = 31, 91%) answered they had heard about OH, while six (8%; without Colombia *n* = 3, 9%) declared that they had never heard about it. When asked to define OH in one sentence, 52 respondents (68%; without Colombia *n* = 16, 47%) included the words human, animal, and environmental health as essential components to define OH. However, the words “intersectoral” and “trans/multidisciplinary/holistic” were used by only 17 people (22%; without Colombia *n* = 11, 32%), 4 named “collaboration/sharing” (5%; without Colombia *n* = 3, 9%). Finally, 15 participants gave definitions that did not align with the WHO definition of OH or appeared off topic (20%) without Colombia *n* = 4, 12%. Examples of these answers were: “*Total health*,” “*Healthy*,” “*Wellbeing for everyone and all in harmony*.”

When participants were asked if they were currently involved in OH initiatives, the large majority stated to be involved (*n* = 52; 68%); this percentage was 77% without Colombia (*n* = 26). Most people involved had studies in “public health” (*n* = 25, 48%) followed by “animal husbandry” (*n* = 24, 46%) and “human health” (*n* = 1, 2%), environmental health and economic sciences (*n* = 1 each, 1%; without Colombia *n* = 1, 4%). On the other side, 24 respondents stated that they were not involved in OH initiatives and most of them (12) belonged to Colombia. Those who declared not to be involved in OH initiatives had a disciplinary studies in animal husbandry (*n* = 13, 54%; without Colombia *n* = 3, 43%), public health (*n* = 7, 29%; without Colombia *n* = 4, 57%), education, human health, animal health, and commerce (each *n* = 1, 4%).

The participants were also asked to briefly describe the OH initiatives. Since this was an open answer, we categorized it in: zoonoses in general (20 answers, 38%; without Colombia: 6 answers, 23%); vector-borne zoonoses (*n* = 7, 13%; without Colombia *n* = 3, 12%); OH without deepening any specific field (*n* = 8, 15%; without Colombia *n* = 3, 12%); antimicrobial resistance (AMR) (*n* = 5, 10%; without Colombia *n* = 3, 12%); animal welfare (*n* = 5, 10%; without Colombia *n* = 1, 4%); education (*n* = 3, 6%; without Colombia *n* = 1; 4%); food hygiene (*n* = 1, 2%; without Colombia *n* = 1, 4%); and chemical safety (*n* = 1, 2%). One Health initiatives on zoonoses were mostly cited by people with an education in “public health” (*n* = 15), followed by “animal husbandry” (*n* = 10), and “human health” (*n* = 1). The respondent with economic background was involved in initiatives on zoonoses (*n* = 1). Education activities were cited by “animal husbandry” (*n* = 2) and “public health” people only (*n* = 1).

Forty-nine respondents stated that OH had been officially endorsed by their institutions, while 7 Institutions did not endorse; 20 respondents said “*No answer/I don't know*.” The institutions endorsed OH by implementing initiatives regarding health education (*n* = 20), research on zoonosis/AMR/vector-borne diseases (*n* = 14), zoonoses in general (*n* = 5), public health and public health policy (*n* = 5), one welfare (*n* = 3).

The respondents cited some examples of programs for which a OH approach was adopted in their institutions. These examples mostly referred to zoonoses surveillance and control (*n* = 9), health education (*n* = 19), research (*n* = 10), and environmental health (*n* = 3). Other cited examples were animal health (*n* = 2), and OH in general (*n* = 2). Antimicrobial resistance and One Welfare were cited once.

When asked to score—from 1 (low) to 5 (high)—some advantages of OH described in literature, respondents appeared to consider all the advantages important. In fact, the median score for “Early detection of threat and timely, effective or rapid response,” “Better/improved/more effective disease control and/or biosecurity measures,” “Improvement in human or animal health or well-being,” “Ecosystem benefit,” and “Design of health policies” was 5. A score of 4 was given to “Economic benefit/increase in economic efficiency,” “Higher quality or larger quantity of information and data and improved knowledge or skills,” and “Personal or social benefits,” being a high score still. This result did not change when Colombia's responses were excluded.

**Figure 3:**
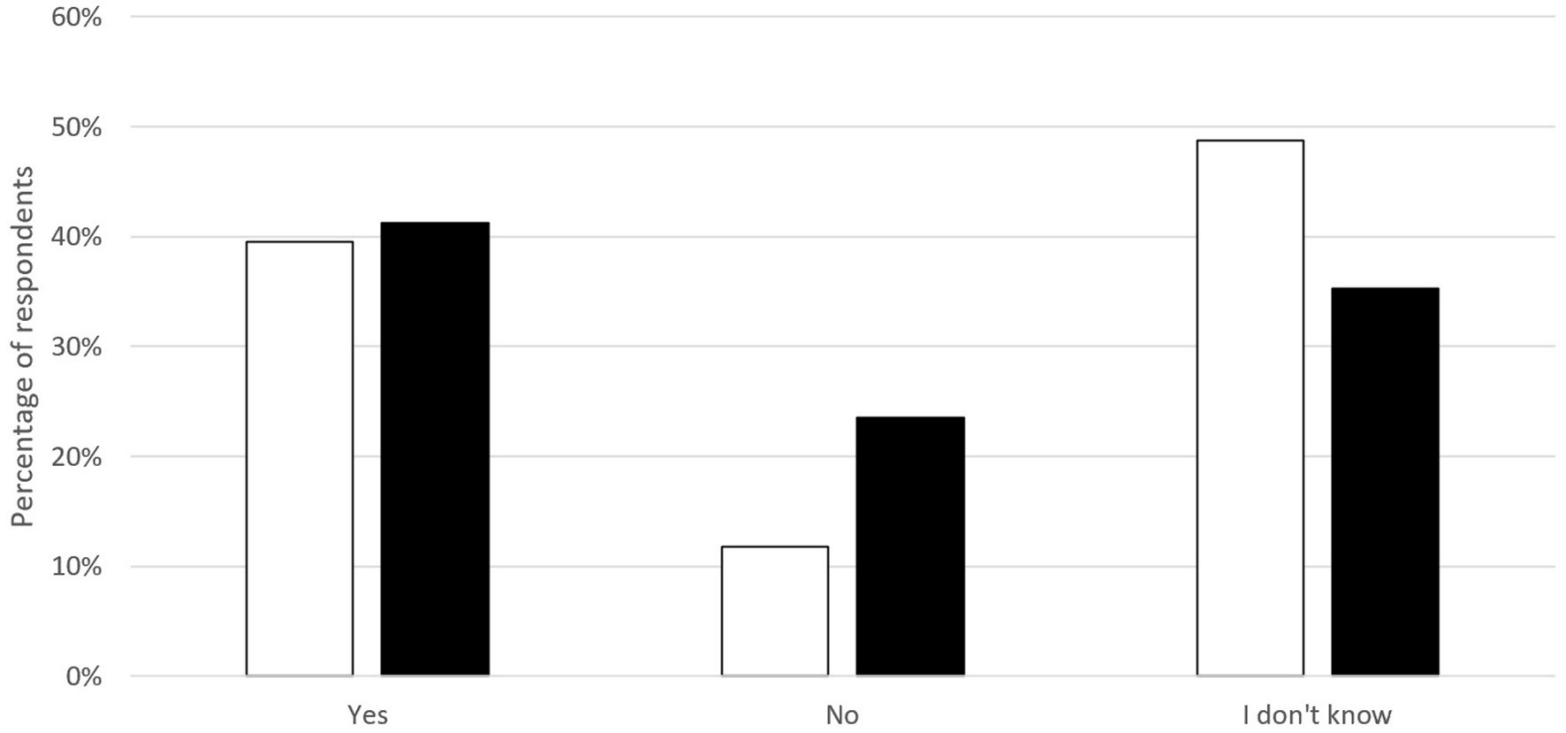
Knowledge about existence of boards/committees/associations actively dealing with One Health issues/initiatives in Latin America; Colombia included (white) and excluded (black).

Around 40% of respondents were aware of the existence of boards/committees/associations actively dealing with OH issues/initiatives in their country (Figure 3). Some respondents provided details, stating boards and networks of Physicians and Veterinarians such as: “Public Health Veterinary Council,” “Rickettsiosis Program and Vector Borne Diseases” in Mexico; “Antimicrobial Resistance Group” in Brazil; “Coordinating Committee for Research in Animal Health” in Uruguay; “Applied Research Center of Chile (Ciachi)” (https://ciachi.org/es/) and “Health Ministry and Academia” in Chile; “Rabies and Brucellosis National Control Programs” in Guatemala; “Animal health and ecosystem” in Argentina; “Sapuvet network” (https://www.sapuvetnet.org/) in Peru; “National Wildlife Veterinary Council” in Costa Rica; and “National Health Institute,” “National Zoonoses Control Program,” “One Health Groups from Academia,” “One Health Network and Food safety” in Colombia.

**Figure 4:**
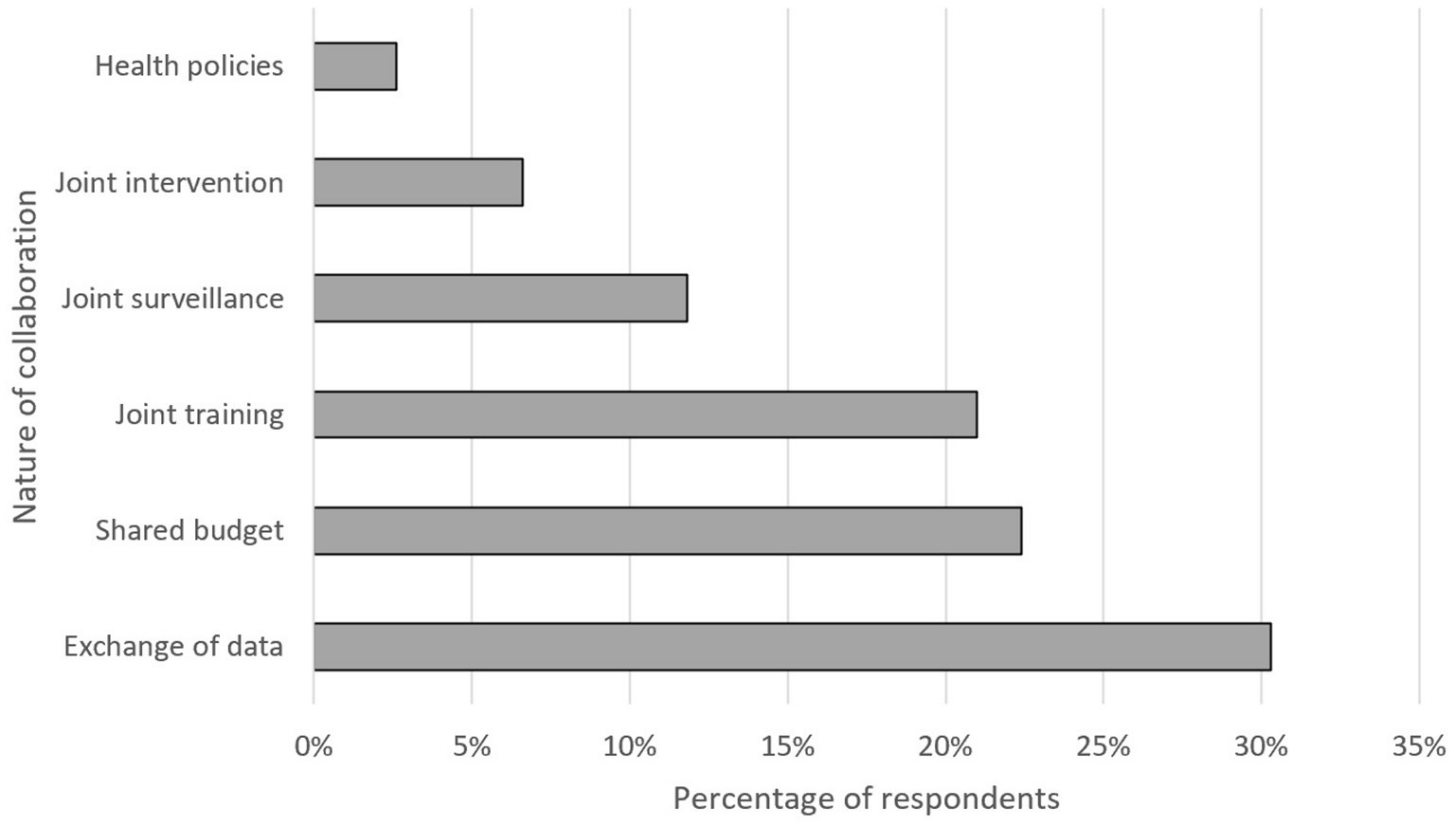
Nature of the collaboration on One Health.

**Figure 5:**
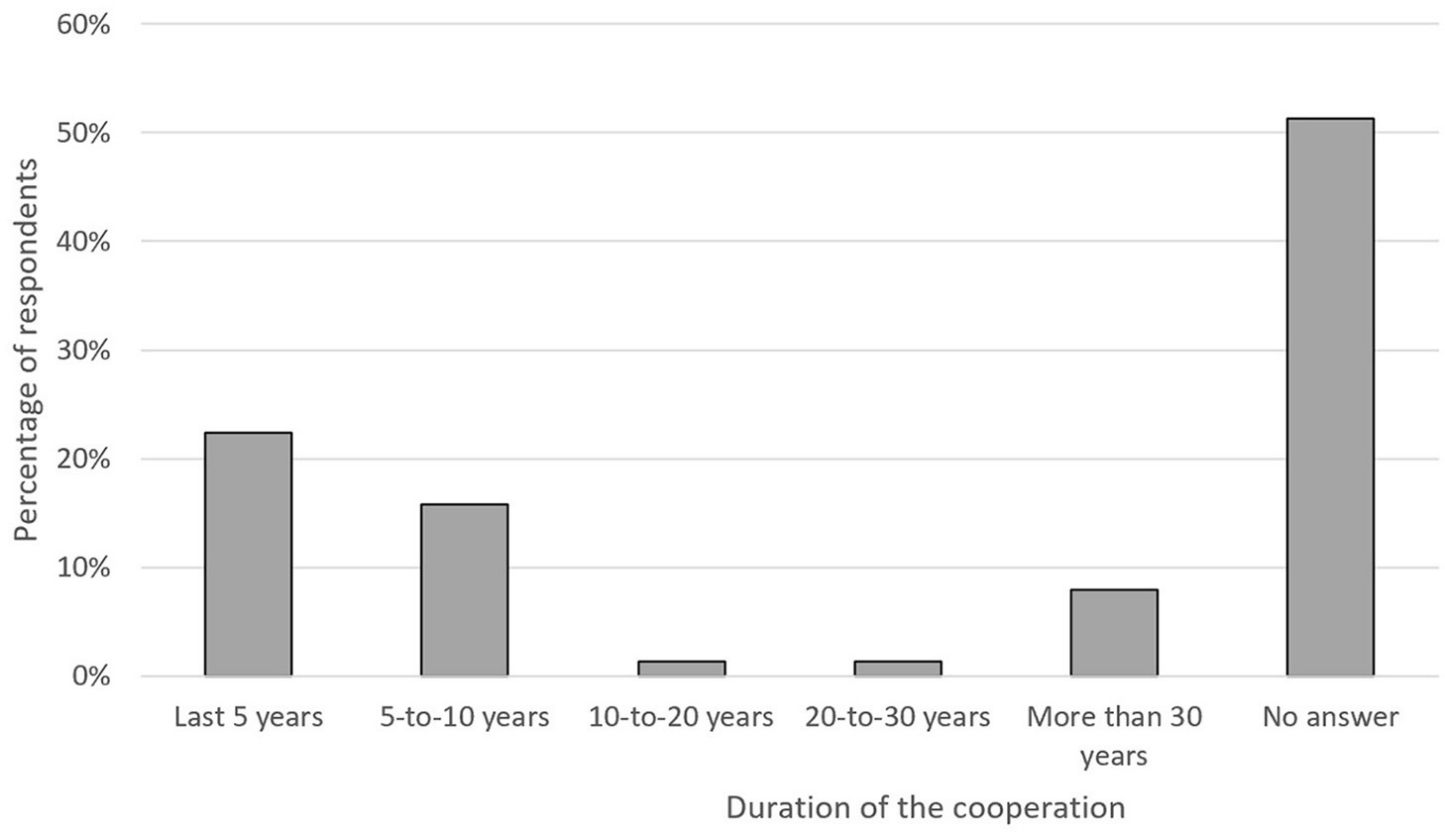
Duration of the cooperation on One Health.

Table 1 and Figures 4, 5 show the respondents' opinion about the level, nature and duration of such cooperation. As shown, the majority of respondents stated the main advantages were the exchange of data, shared budget, and joint training. Likewise, most of the respondents described the duration of those initiatives to be <10 years (38%). Half of the respondents was not aware of the duration of the OH initiatives.

**Table 1:** Level of connections on One Health.

	**AR**	**BO**	**BR**	**CR**	**CH**	**CO**	**EC**	**GT**	**MX**	**NI**	**PE**	**UR**	**VE**
National	17.6%	0	5.8%	5.8%	5.8%	35.3%	0	0	17.6%	5.8%	5.8%	0	0
National-Subnational and local	33.3%	0	0	0	0	66.6%	0	0	0	0	0	0	0
National and subnational	0	0	0	0	12.5%	75%	0	0	12.5%	0	0	0	0
National and local	0	0	0	0		0	0	0	0	0	0	100%	0
Subnational	0	0	0	20%	0	60%	0	0	0	0	20%	0	0
Local	0	0	0	0	0	83.3%	0	0	16.6%	0	0	0	0
Subnational-local	0	0	0	0	0	50%	0	0	0	0	0	0	50%
I don't know	2,5%	5.1%	2.5%	0	2.5%	56.4%	5.1%	2.50%	10.2%	2.5%	7.7%	0	2.5%

*AR, Argentina; BO; Bolivia; BR, Brasil; CR, Costa Rica; CH, Chile; CO, Colombia; EC, Ecuador; GT, Guatemala; MX, Mexico; NI, Nicaragua; PE, Perú; UR, Uruguay; VE, Venezuela*.

Twenty-five respondents were aware of 1–5 OH initiatives being implemented, three people indicated 6–10 initiatives, and four respondents more than 10 initiatives. Thirty-nine respondents did not know in which field these initiatives were implemented. Other respondents cited disease surveillance and monitoring (*n* = 33), disease prevention and control (*n* = 34), research (*n* = 28), participants' awareness on the programs (*n* = 19), and higher education programs (*n* = 20). “NextCap” Project in Bolivia and Applied Research Center of Chile (Ciachi) in Chile were cited as local examples of OH projects or programs.

### Functional Cooperation in Zoonotic Diseases, Environmental Health, and AMR

**Figure 6:**
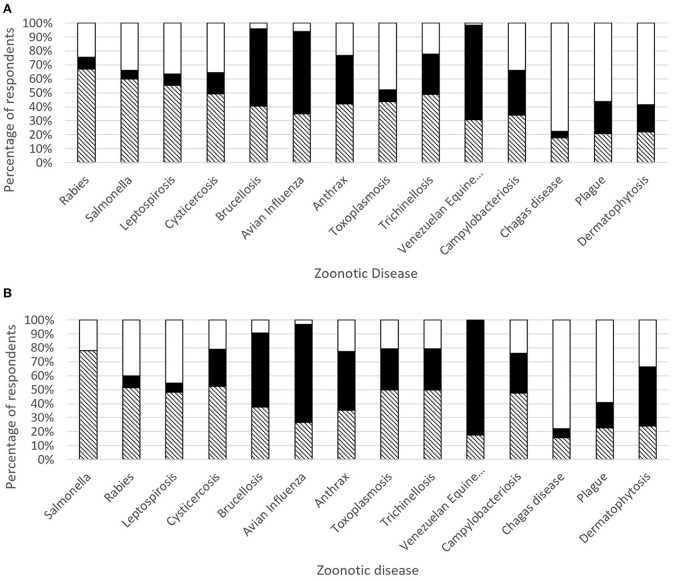
**(A)** Zoonotic diseases control and monitoring by the Ministry of Health (MoH) in white, Ministry of Agriculture (MoA) in black, and both in black-white stripes in Latin America. **(B)** Zoonotic diseases control and monitoring by the Ministry of Health (MoH) in white, Ministry of Agriculture (MoA) in black, and both in black-white stripes in Latin America (Colombia not included).

Twenty-nine people (38%; without Colombia 18 respondents, 53%) claimed that in their countries there is a functional cooperation between the MoH and the Ministry responsible for Animal Health, facing zoonoses. Twenty-two participants 29% answered “no” (without Colombia: 15 respondents, 44%), and the other interviewees answered “I do not know.” Figures 6A,B illustrate which zoonotic diseases are controlled and monitored by the MoH and/or Ministry of Agriculture (MoA) in Latin America (Colombia included and excluded), according to respondents.

Some questions sought to interrogate about the level of knowledge in the community about diseases of animals exposed to environmental pollutants and subsequently transmitted to humans by food of animal origin (e.g., dioxins, PCBs, DDT, and related pesticides). Sixty-five respondents (85%) gave a median score of 2 (Q1–Q3: 2–3) to the level of knowledge about these diseases (scoring from 1—poor, to 5—excellent); nine persons said they were not competent in the field. The median score given to the “quality of national plans for the prevention and monitoring of foodborne diseases of animal origin caused by environmental pollutants” by 60 respondents was 3 (Q1–Q3: 2–4). In this case, 16 people responded they were not competent in the field. The results were not altered by withdrawing the respondents from Colombia.

**Figure 7:**
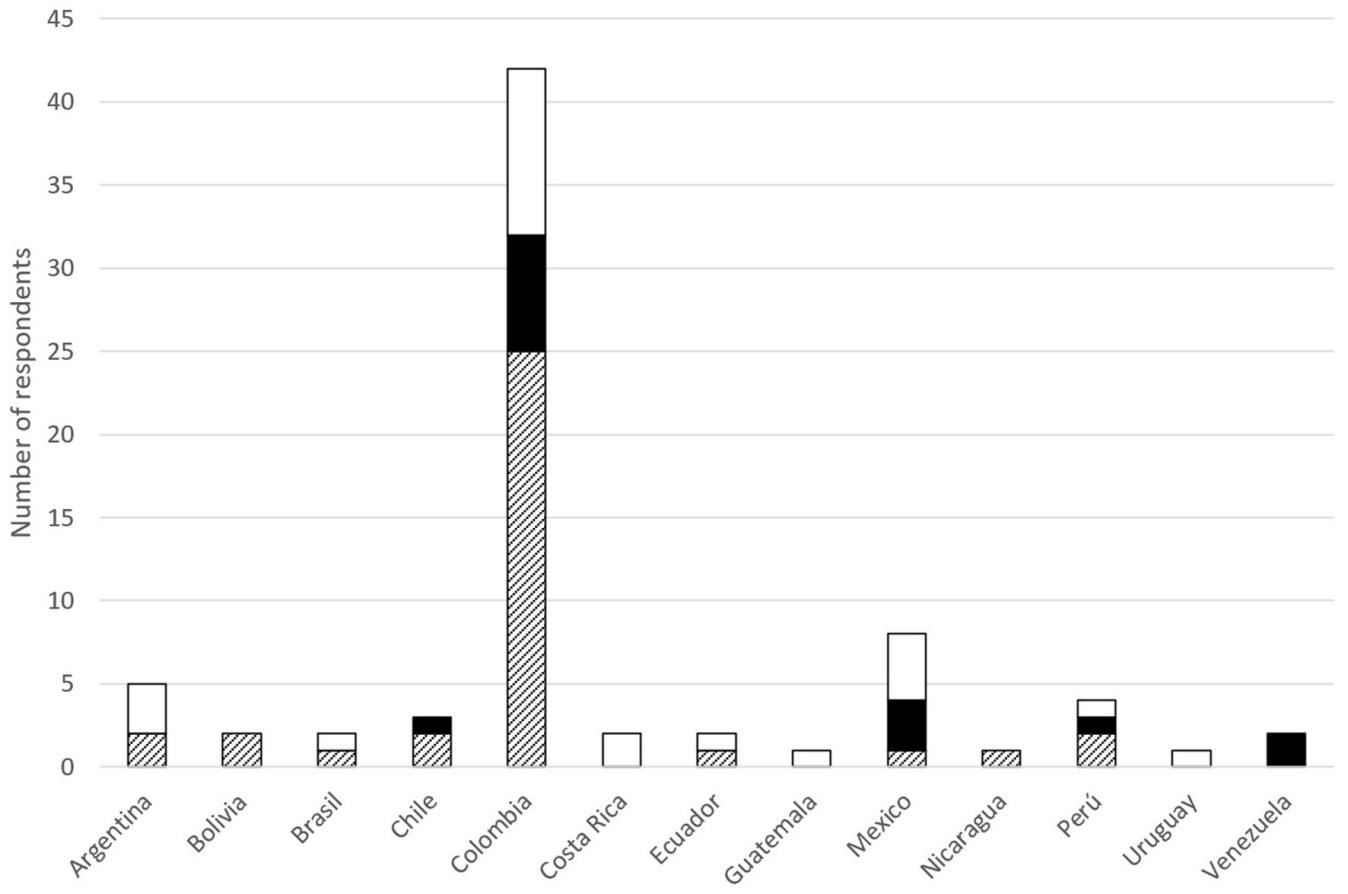
Contribution to Latin America AMR monitoring/research programs, yes (white), no (black), NR (black-white stripes).

Regarding the issue of AMR surveillance in Latin America, with specific monitoring and research programs, 25 respondents (33%; Colombia excluded *n* = 15 respondents, 44%) declared that their countries contribute to that. Thirteen respondents answer “no” (17%; Colombia excluded *n* = 6%). The other respondents (*n* = 38, 50%; Colombia excluded *n* = 13, 38%) did not answer or did not know (Figure 7).

### Factors Limiting Interdisciplinarity and Intersectoral Collaboration

As regards the aspects limiting interdisciplinarity and intersectoral collaboration, the main limit, cited by 27 respondents (36%; Colombia excluded *n* = 17 respondents, 50%), was a “siloed approach” of disciplines, followed by “institutional limits” and “limits on education” cited by six persons each (8% each; Colombia excluded *n* = 2), and “lack of resources” (*n* = 2, 3%; Colombia excluded *n* = 2). Ten respondents mentioned more than one limit. Interestingly, 25 persons (33%) did not answer (Colombia excluded: *n* = 13).

### Perception on the Level, Opportunities and Implementation for One Health Collaboration

Table 2 shows how the participants perceive the level and the opportunities for OH collaborations within professional boards, University Departments, institutions involved in veterinary surveillance and food security, and institutions involved in emergencies management for both groups. Most respondents agreed that the opportunities for collaboration in all the scenarios described above are poor.

**Table 2:** Perception of the level and the opportunities for OH collaborations within several professional scenarios in both groups (all countries and Colombia excluded).

**Level and opportunities for OH collaboration within**.	**Poor (%)**		**Fair (%)**		**Good (%)**		**Excellent (%)**		**n/a (%)**	
	**All countries**	**Colombia excluded**	**All countries**	**Colombia excluded**	**All countries**	**Colombia excluded**	**All countries**	**Colombia excluded**	**All countries**	**Colombia excluded**
*Professional boards*	36 (47.4%)	16 (47.0%)	17 (22.4%)	7 (20.5%)	11 (14.5%)	4 (11.8%)	3 (3.9%)	2 (5.8%)	10 (11.9%)	5 (14.7%)
*University Departments*	23 (30.3%)	11 (32.3%)	17 (22.4%)	9 (26.5%)	19 (25.0%)	6 (17.6%)	8 (10.5%)	3 (8.8%)	10 (11.9%)	5 (14.7%)
*Institutions involved in vet surveillance and food security*	31 (40.8%)	13 (38.2%)	24 (31.6%)	12 (35.3%)	14 (18.4%)	5 (14.7%)	1 (1.3%)	1 (2.9%)	7 (19.2%)	3 (8.8%)
*Institutions involved in emergencies management*	32 (42.1%)	14 (41.2%)	23 (30.3%)	11 (32.3%)	12 (15.8%)	3 (8.8%)	1 (1.3%)	1 (2.9%)	9 (11.8%)	5 (14.7%)

**Figure 8:**
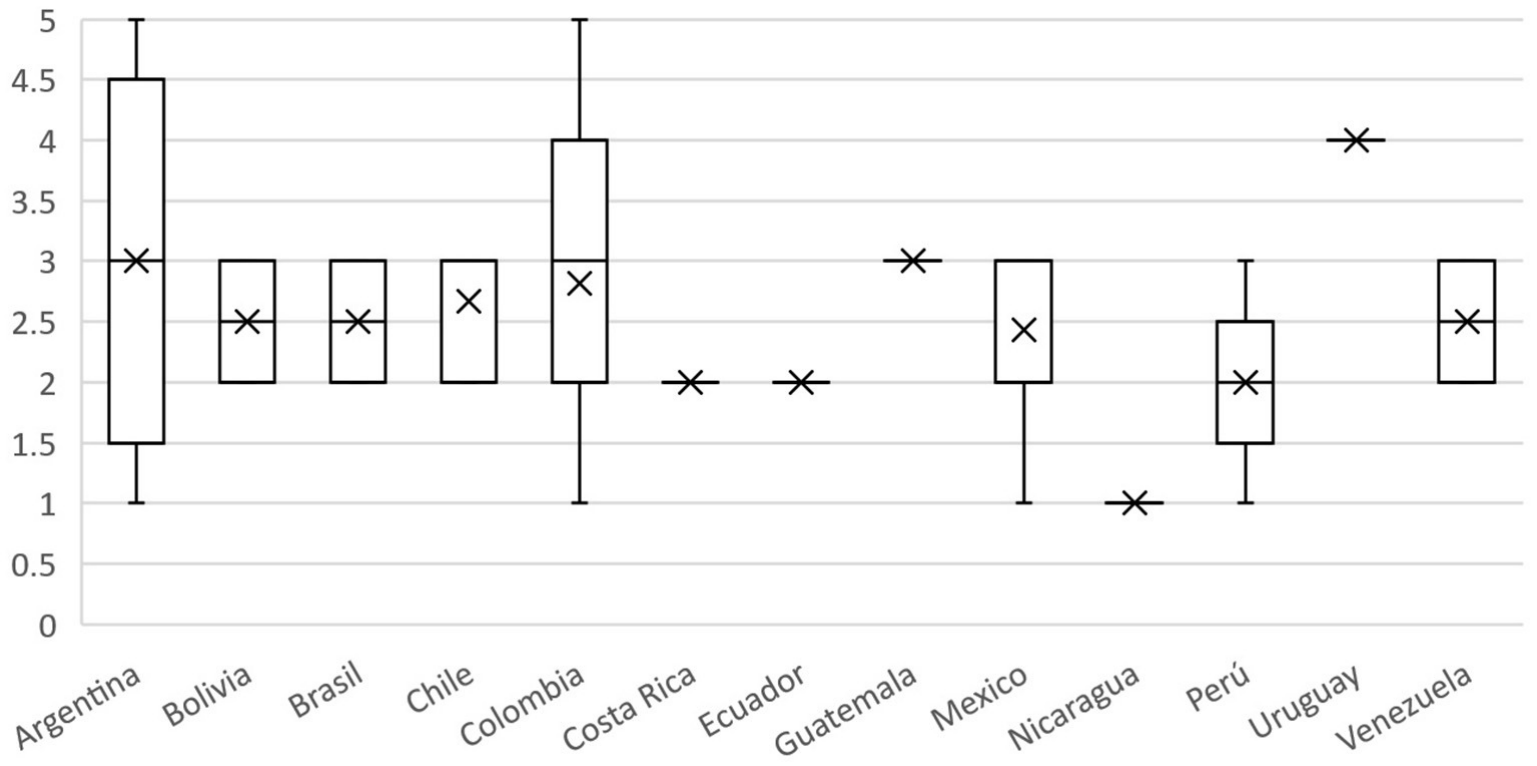
Boxplot of the scores attributed by respondents on the implementation of the OH approach by professionals in their respective countries; scoring from 1 (poor) to 5 (excellent).

Respondents were asked to rate how well the OH approach is implemented by the professionals employed/engaged in Veterinary, Public, and Environmental Health sectors in their country, scoring from 1 (poor) to 5 (excellent). Seventy respondents gave a median score of 3 (without the results from Colombia, the median score was 3 as well). Details of the answers by countries are illustrated in Figure 8. The box-plot illustrates the median score (plus IQR and min/max) attributed by respondents.

Eighteen respondents (24%; Colombia excluded 7 respondents, 21%) asserted the existence of recent formal initiatives to establish and/or to strengthen intersectoral collaboration with the objective of working with a OH approach. Twelve people (16%) answered “no”; the other respondents selected “not answer/I don't know.”

### Examples of “Burning” OH Issues/Initiatives

**Figure 9:**
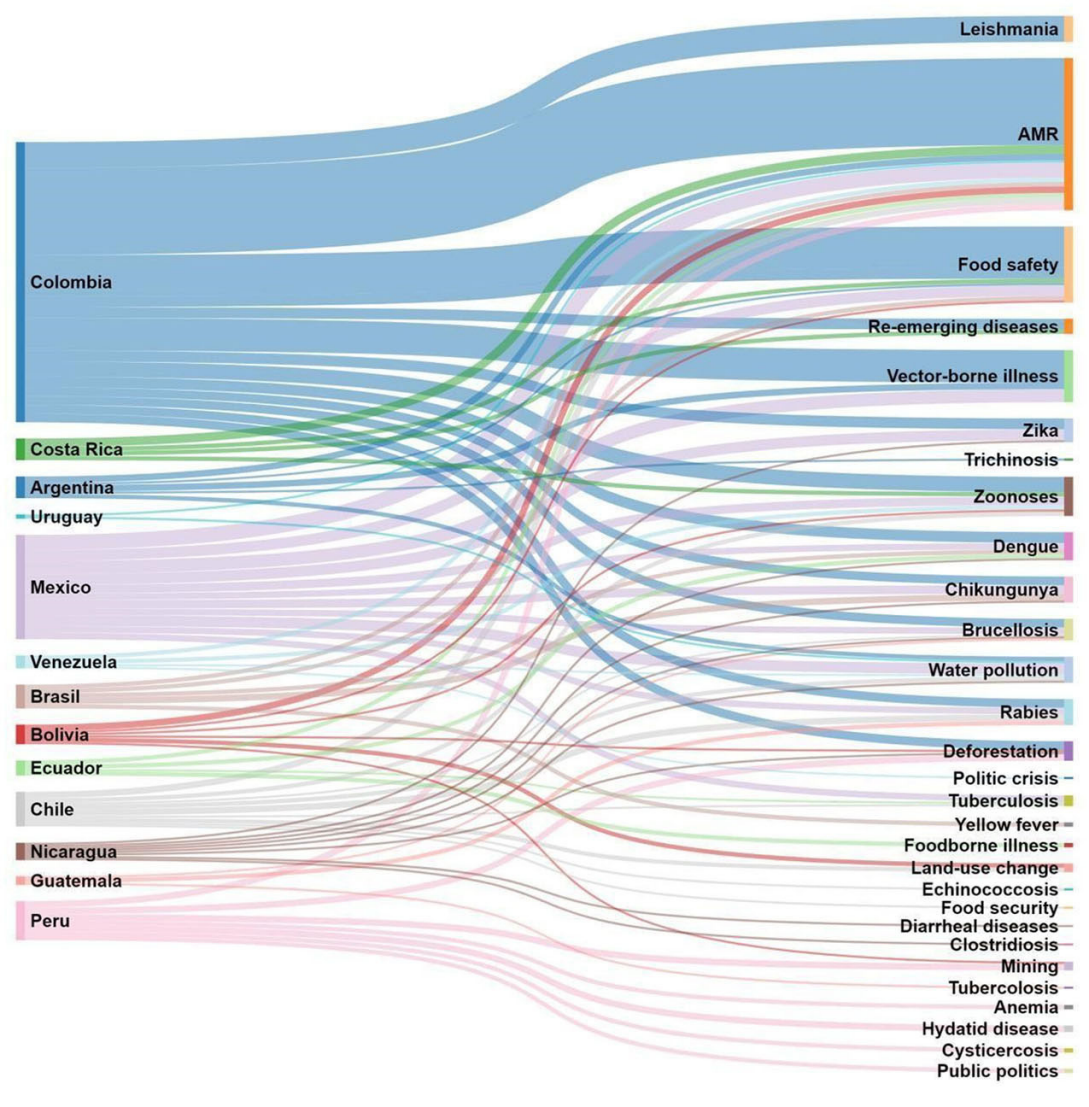
Top environmental, animal, and human health issues over the past 5 years cited by countries in the different regional areas (Colombia excluded).

The participants were asked to cite the top three environmental, animal, and human health issues in their country over the past 5 years. The vast majority (92%; Colombia excluded: 97%) cited AMR, food safety (*n* = 56, 74%; Colombia excluded: 47%) and zoonoses (*n* = 12, 16%; Colombia excluded: 43%). The Sankey diagram shows all answers by country (Figure 9). When considering answers by countries in the same geographical region, we observed differences in the top three issues: AMR was cited in all countries except from Guatemala; food safety in all countries except from Ecuador, Chile, Nicaragua, Guatemala, and Peru; all except Nicaragua, Guatemala, and Peru cited zoonoses.

### Gaps in One Health Approaches

Gaps in OH plans were identified as the “siloed approach of disciplines or lack of articulation among sectors” (*n* = 26, 34%), “government barriers/lack of political will and laws to create synergies” (*n* = 18, 24%), “barriers for OH communication/lack of education of OH approach among institutions and citizens” (*n* = 9, 12%), and “lack of resources and budget” (*n* = 11, 14%). Other breaches cited by fewer respondents were that priorities are focused on human health but not on animal and environmental health (*n* = 3, 4%) and the institutional corruption (*n* = 1, 1%). Twenty-three (30%) of the respondents did not answer. From those 23 people, their discipline were animal sciences (*n* = 11), public health (*n* = 8), human health (*n* = 1), commerce (*n* = 1), health education (*n* = 1). From the 23 respondents who did not answer, 13 were from Colombia. When removing Colombia's answers, the results were the same.

**Figure 10:**
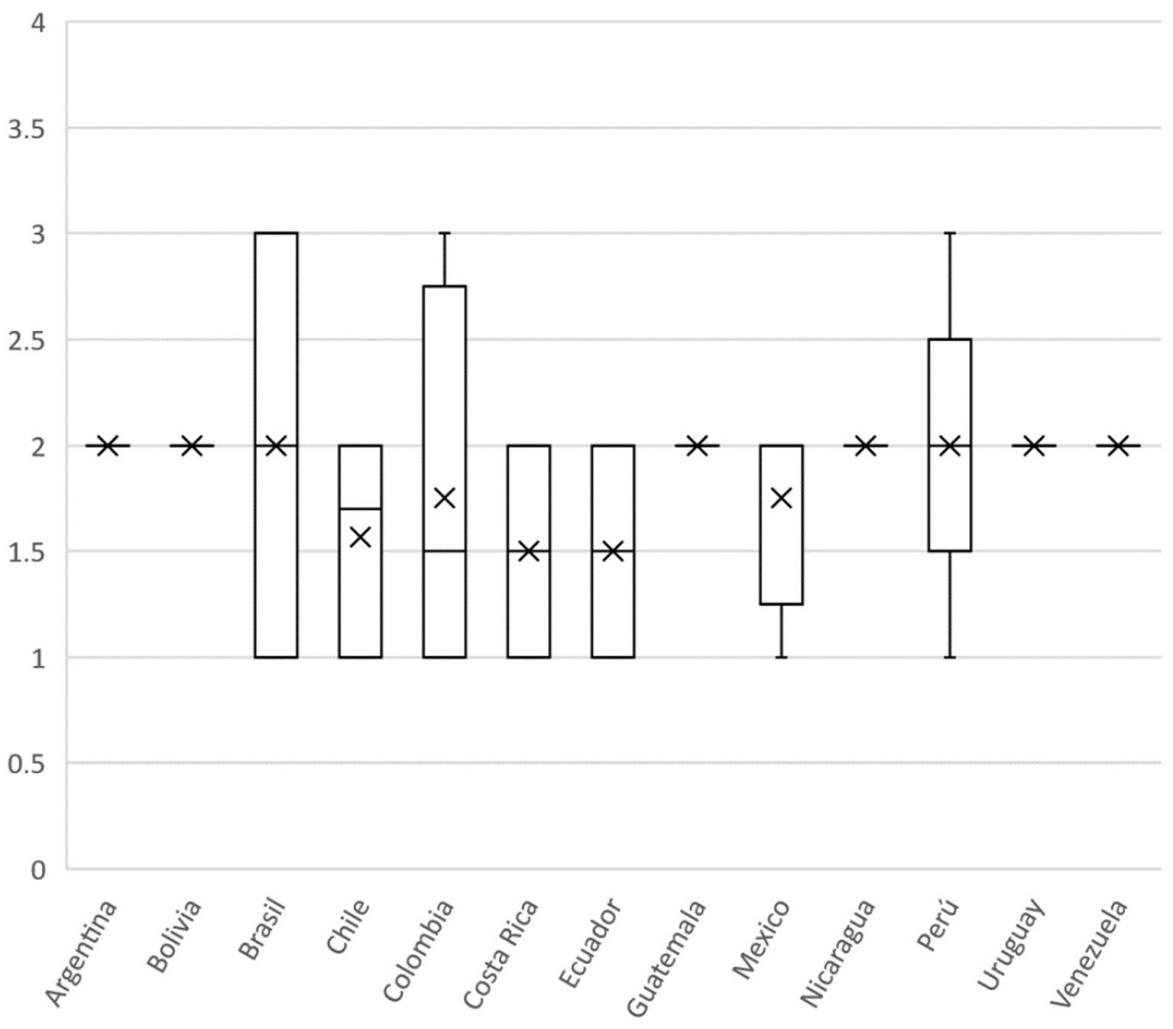
Boxplot of the scores attributed by respondents to the level of awareness on OH in citizens in their respective countries; scoring from 1 (poor) to 4 (excellent).

According to participants, the level of knowledge/perception of OH amongst citizens/consumers in their country is very low. In fact, the median score was 2 (Q1–Q3: 1.75–3.0) in a range from 1 (poor) to 4 (excellent). Even if we removed Colombia's answers, the median score remained 2. Details of the answers aggregated by countries are illustrated in Figure 10. The box-plot illustrates the median score (plus IQR and min/max) attributed by respondents.

Only some respondents added a few comments, remarks and suggestions to the questionnaire: … “*there is a certain apathy from those responsible for human health to integrate animal health professionals into a conjoint work,”* “…. *hopefully the actions in favor of OH will be a priority because of the Pandemic”*…”*Our countries must: 1. receive greater commitment from government institutions. 2. Strengthen training for communities and unions. 3. Include lines of training on One Health in formal primary, secondary and university education programs, as well as in informal training programs* “…” we *should include requirements for OH in health sector legislation, including specific budgeting of resources....*”

## Discussion

There is currently no record in Colombia or Latin America that would allow understanding of the OH baseline on perception, knowledge, and barriers among main stakeholders.

### General Information

The vast majority of respondents to our survey had a background in animal health and public health, and slighter engagement from the environmental component of OH. Education, commerce, economics, biology, and evolution disciplines 1% each. Chiesa et al. (16), obtained similar results in the European study, where the majority of respondents declared to had training or professional studies in animal health or animal husbandry (54%), followed by Public Health or Human Health (30%), and only 10% of respondents had Environmental Sciences studies. In addition, most of our respondents worked at higher education institutions/universities followed by governmental institutions/ministries, research centers, NGOs, and the private sector. In the European study, a lower percentage of interviewees worked at Higher Education Institutions/Universities and in NGOs, while there was a higher participation from Governmental Institutions/Ministries, research centers, and private sector. The larger proportion of respondents from animal health, as well as academic and research institutions can be explained by the fact that, in Latin America, OH was first made known in universities with veterinary medicine schools through the Sapuvet network in the 2000s. Since then, the OH concept has been promoted strongly by animal health academy and research communities (8, 13, 14). Also, the European study was carried out during the framework of action of the Network of Evaluation of OH, while in our study the survey was done independently by the initiative of Academia without predetermined resources and Government collaboration.

### About One Health

“One Health” was a familiar concept for the majority of respondents (92%). Overall, 68% of respondents mentioned the words human, animal, and environmental health as essential components to define OH. However, the words “intersectoral” and “trans/multidisciplinary/holistic” were used by only 22% (excluding Colombia 32%), and only 5% (without Colombia 9%) mentioned the aspect of “collaboration/sharing.” Our results differed moderately from the European study (16), higher percentages of respondents included a term among “intersectoral/transdisciplinary/holistic” in the definition and named “collaboration/sharing.” This may be explained by the fact that the OH approach has been studied the most by animal health and public health (21), so a traditional understanding of OH evolving around the linkages between “human,” “animal,” and “environment” health exists. Only 20% of interviewees did not answer properly or gave an incomplete or unclear definitions, indicating that one-fifth have a lack of understanding of the concept among the knowledgeable audience. This is in accordance to Xie et al. (22), who stated that, despite the OH concept's growing popularity and acceptance by the professional community, the definition of the term remains imprecise. It is important to highlight that the environmental health component was mentioned frequently, in 68% of the answers, which suggests that the three pillars of OH are overall perceived as having equal importance. In contrast, in Europe, only 42% of the respondents mentioned the environment (16) showing that in Latin America the environmental component is taken more into account.

The background of respondents who claimed to work on zoonoses was mostly public health and animal science. The high frequency of people joining OH initiatives on zoonoses is reflected by the significant amount of literature in OH describing the importance of the approach in the control and prevention of zoonoses. Moreover, the major contributions to improve our understanding of complex health relationships and to reduce national and global health risks are carried out on zoonoses topics (20, 23–25). Thus, in order to gain a more in-depth understanding of the socio-economic and ecological determinants of human, animal, and ecosystem health, the OH approach is the most promising way for dealing (prevent and control) with multi-scale, system-wide threats such as pandemics. Regarding that, the United Nations Environment Programme (26) stated that more investment and support is required before such approaches can be implemented routinely. Thus, a standardized set of metrics to measure the effectiveness of OH interventions on zoonoses may also help to increase uptake of the approach (26). In this sense, a National Program for the Integrated Control and Prevention of Zoonoses based on OH approach was designed in Colombia to support the policy decision making for zoonotic diseases in 2016. Lessons learnt from that experience showed that active integrated cooperation to prevent and control zoonoses is adopted only in outbreak situations or public health emergencies but not as continuum systematic way of working among sectors (27). Furthermore, there are limits to data sharing, joint cross-sectoral coordination mechanisms and joint risk assessment among Ministries and a shared budget to implement OH activities or priorities is absent. Despite that, Colombia was pioneer in holding the first CDC-OH Zoonotic Prioritization workshop in Latin American as an example of a collaborative and joint simulation exercise in 2019 (28), providing a good model to engage countries in the OH reflection.

The major advantages and outcomes of OH were identified by our respondents as the early detection of threat and timely, effective or rapid response, better/improved/more effective disease control and/or biosecurity measures, improvement in human or animal health or well-being, ecosystem benefit, and design of health policies. This indicates the importance of OH when dealing with outbreaks from animal origin and when assuring integrated health policies. Lower scores were attributed to other aspects (“Economic benefit/increase in economic efficiency,” “Higher quality or larger quantity of information and data and improved knowledge or skills,” and “Personal or social benefits”), corroborating the lack of evidence of the added value in economic and research aspects. In Latin American countries, the social, economic, environmental advantages of implementing OH as a way for better health governance is still a long way to go, given the priorities generated by the huge social inequalities.

The fact that about 40% of respondents reported to know formal connections, committees, or initiatives of OH in their country is valuable. However, most of respondents denied or ignored the existence of OH initiatives in their countries (Figure 3), which indicates the lack of true integration of activities in field. Several projects and activities have been developed and are now working within the OH concept at the national, regional, and global level, as mentioned in the results, based on the expectation that a more holistic management of microbial health hazards will result in a more efficient use of the scarce resources available for mitigating zoonotic disease risk (20, 24–26). However, such a paradigm shift has not been supported by the systematic allocation of resources to integrated national or multinational programs. As said before, at the national level, Ministries of Health and Agriculture (or Animal Health) remain largely separate, with individual budgets and agendas (20).

The number of interviewees involved in OH initiatives that belonged to “environmental health” sector was low. We acknowledge the potential selection bias in our study because the participation of the environmental sector was limited, although we attempted to contact professionals from this area. However, De Freitas (29) reported that in Latin America, the environmental dimension (ecosystem) has never been taken into account in a systematic way, therefore environmental health professionals do not tend to participate in intersectoral work (29). This could also explain the fragile collaboration between professionals in human health, animal health, and in the ecosystem areas (29). This happens despite six of the countries with the world's greatest biodiversity are found in Latin America: Brazil, Colombia, Ecuador, Mexico, Perú, and Venezuela. This region is also home to the habitat with greatest biodiversity in the world (30). Even if the participation of the environmental professionals was low, almost all respondents (90.8%) considered environmental health a OH pillar.

### Zoonotic Diseases, Environmental Health, and AMR: Examples of “Burning” OH Issues/Initiatives

The examples of OH issues/initiatives provided, showed interesting insights. Only 38% of the respondents reported an active cooperation in their countries between the MoH and the Ministry responsible for Animal Health (MoA), when dealing with zoonoses, also stating that there is an obligation to guarantee a reciprocal flux of information between Public Health and Animal Health services. The wildlife diseases that are present in Latin America were not explicitly addressed in our list, because these diseases are underreported and wildlife research is not as closely connected to domestic animals and humans. We know there is an information gap produced by the lack of well-established bodies and surveillance programs for the wildlife diseases, we included only those with recognized surveillance in Latin America countries.

Our study showed that respondents gave importance to classical endemic zoonoses as well as emerging zoonoses as they stated they should be monitored and controlled by both, MoH and MoA. Diseases like rabies, salmonellosis, leptospirosis, cysticercosis, brucellosis, avian influenza, anthrax. In Sankey diagram we noticed that other infectious zoonotic diseases are cited such as: trichinosis, tuberculosis, echinococcosis, and the vector borne diseases (VBD) like zika, chikungunya, dengue, leishmaniasis, yellow fever, indicating that environment aspects should be considered in control and prevention. These results are in accordance with local authors in Colombia who pointed out that influenza A (H1N1), leptospirosis, brucellosis, rabies, and toxoplasmosis are the zoonoses with high priority in 2012 (31). Likewise, in Brasil, Gonçalves, et al. (32) reported that Lyme diseases, brucellosis, leptospirosis and toxoplasmosis are related to the low social, economic and cultural conditions of the population from small rural properties have resulted in lack of basic information on animal health and direct or indirect contact with the various species of domestic animals, wildlife and ticks have probably contributed to the prevalence levels found. The presence of such diseases is seen in marginalized populations, reflecting the lack of equity in our society and the lack of attention to the social determinants of health (SDH) and risk factors (33, 34). Neglected infectious diseases in Latin America are often left out as public health priorities, or their prevention and control programs are underfunded or are deemed unsustainable. The SDH are especially important in Latin American countries, which are characterized by adverse colonial legacies, tremendous social injustice, huge socioeconomic disparities, and wide health inequities (34). Poverty and inequality worsened substantially in the 1980s, 1990s, and early 2000s in these countries (33). Many Latin American countries have introduced public policies that integrate health, social, and economic actions, and have sought to develop health systems that incorporate multisectoral interventions when introducing universal health coverage to improve health and its upstream determinants (33). However, these conditions and factors continue to be present in most of these countries, and a clear long term solution is still needed. Health inequalities and inequities throughout the Americas are persistent and manifest through the occurrence of these diseases, providing crude illustrations of severe deprivation, and misery in vulnerable populations (34). Regarding the complexity of the surveillance, control, and prevention of zoonoses, the need to implement integrated epidemiological surveillance systems for these classical zoonoses in both animal and human health is critical. Important efforts are needed to improve the lack of information on zoonoses due to the poor regional surveillance systems (35). The underreporting of zoonoses in human health has been explained in Colombia by the indifference from the medical doctors about zoonotic diseases, and by the logistic and institutional barriers for laboratory confirmation of those disease (36). Many factors contribute to underreporting of zoonoses, arising from both an inability and an unwillingness to report. The relative importance of these factors varies in different situations, but they often act in combination to stifle the collection and distribution of accurate and comprehensive data, particularly in resource-poor settings (37). In the area of clinical practice, medical schools do not typically emphasize the ecology of microorganisms; so, medical students do not see the importance of zoonotic diseases and the impact on human health, and therefore, they do not see the need to work with their veterinary medical colleagues. This evident gap finds its beginnings from the education of the medical school, where the focus is only on the human being. Contrary to that, veterinary medicine seeks to teach students about different species, including humans, which allows a more obvious will to collaborate with other areas (38).

The Sankey diagram (Figure 9) shows that the most frequent health problem mentioned in Latin America was AMR. However, 50% of the participants stated, answering another question in the survey (Figure 7), that they did not know if their respective countries contribute to AMR surveillance with specific monitoring and research programs. To the authors' knowledge, AMR surveillance is one of the best examples of the impact of the OH approach in practice in the Region. Indeed, since 2010, there has been a strong commitment from FAO, OIE, and PAHO, working together to mitigate the risks of AMR in the interconnection among human health, animal health, and the environment. With participation of representatives of Ministries of Health and Agriculture from Argentina, Brazil, Chile, Colombia, Paraguay, Peru, and Uruguay, the organizations now joined forces in the implementation of the project “Working Together to Fight Antimicrobial Resistance” to ensure a coherent “One Health” approach, recognizing the multidimensionality, and necessity of an intersectoral response that is needed to address the problem of AMR (39). Among the 25 respondents (33%) who were aware of AMR initiatives in their countries, only 6 (from Colombia and Ecuador) mentioned the FAO, OIE PAHO initiative described above. Other initiatives described were from Argentina, Chile, Perú, Brazil, Uruguay, but the names of the programs or projects were not mentioned. It is perplexing that none of the participants from Colombia, mentioned the Colombian Integrated Program for Antimicrobial Resistance Surveillance (COIPARS), a program created for AMR surveillance in poultry farms that was the first initiative to explore the implementation of OH (40).

### Aspects Limiting Interdisciplinarity/Intersectorality in OH

In the section regarding the aspects limiting interdisciplinarity and intersectorality in OH (section 4), the “siloed approach” of sectors, followed by the siloed approach of disciplines, was the most commonly mentioned limiting factor (34%). This factor has long been recognized as a barrier to moving toward OH by several authors worldwide (41–43). Johnson et al. (41) reported that the absence of a clear definition and subsequent vision for the future of OH act as a barrier to interdisciplinary collaboration, and that siloed approaches/lack of communication by different sectors restrict the ability for professionals to work collaboratively across disciplines (41). In the same way, Manlove et al. (42) stated that efficiently disseminating knowledge and methodologies across disciplinary boundaries is essential for a cohesive reaction to emerging threats. However, researchers tend to organize themselves into discipline-specific “silos” that contain robust internal research communities, but that only rarely interact with one another. This is particularly true of the disciplines studying infectious disease: workplaces range from hospitals, to microbiological laboratories, to ecological field sites, to mathematical computing facilities, and communicating across these physical and cultural boundaries is difficult (42). Likewise, Nyatanyi et al. (43) reported the Rwanda's government need to fund the implementation and embrace the concept of “oneness,” such that the separate ministries can develop common policies, approaches and evaluations that can feed into action plans and improved health infrastructure. Academics also need to think beyond the traditional silos (medicine, public health, veterinary medicine, engineering, etc.) in ways that will stimulate innovation and encourage problem solving (43).

Concerning the other gaps that emerged from our study, the general low awareness about OH, lack of implementation about OH, lack of commitment of policy-makers, resources, and budget for OH. Chiesa et al. reported similar results in their study in Europe (16). We compared our findings with the classification reported by Ribeiro et al. (44). They offered the challenges and difficulties for executing OH initiatives in the following three categories: 1. *Conditions for starting*: policy and funding; education and training; 2. *Execution*: surveillance; multi-actor, multi-domain, and multi-level collaborations; and 3. *Monitoring and evaluation*: evidence (44). Based on this classification, several barriers were cited in our study as follows in policy and funding: “*lack of funding, normative and inclusion of research results within the Governmental sector, “low political will,” “personnel reluctant to change*.” On the education and training, obstacles were cited as follows: “*lack of awareness on these topics from the human health sector,”* “*insufficient training programs on OH concept and application*.” Referring to the surveillance level, one respondent answered: “*logistical challenges such as lack of personnel supporting the environmental component in national programs*” “*Need of diagnostic laboratory capacity for wildlife,”* “*ambiguous legislation for integrated surveillance across different domains (environmental, animal, and human health systems,”* “*Restricted access to data, conflict of interest, selfishness, and lack of interest on those topics*.” On the multi-actor collaboration and multi-domain collaboration, difficulties were described as: “*the little opening of each sector for collaborative work,”* “*Sectors work in isolated way,”* “*difficulties in promoting the engagement of multiple actors across domains*.” Regarding the multi-level collaborations, problems were cited as: “…*institutional corruption*…*Colombians have unhealthy practices in the search of resources to maintain their families due to social inequities” “Professional egos hindering the intersectoral collaboration*.” Dos Ribeiro et al. (44) reported the lack of OH evaluation studies and reporting of outcomes and lack of guidelines and metrics for OH monitoring and evaluation but in our study, we did not find any answer about these specific challenges.

As regards the perception of the level and the opportunities for OH collaborations within several professional scenarios (Table 2) the “poor” scores prevailed as regards professional boards, institutions involved in animal surveillance and food security and institutions involved in emergencies management. This result suggests that in Colombia and the other participating countries there is an overall negative perception about OH collaboration, despite its potential benefits. According to our data, there is a common understanding that OH is beneficial to design and implement better public health programs, but the implementation of the OH approach remains a huge challenge (Figure 8). One Health implementation is qualified between *insufficient* and *limited* in all participating countries. This is in accordance with Yamada et al. (45), who pointed out that OH operationalization has so far proved to be challenging. Implementation is often a complex issue requiring collaboration between diverse and multi-disciplinary partnerships (45). At a local or national level, it often might be a matter of breaking down professional barriers through improved communication and incorporating information on OH and its benefits into professional training and university courses. At the international level, it is usually much more difficult and can be hindered by dysfunctions which characterize current forms of global health governance (45). Regardless of the gaps and barriers mentioned by participants for the OH implementation, Pettan-Brewer et al. (46) reported that local communities from diverse social and economic status, including indigenous populations, have been working with institutions and social organizations for many years, especially in Brazil, accomplishing results through grassroots movements. These “bottom-up” socio-community approaches, have been also tools for prevention and control diseases (46).

### Limitations of This Study

Although the questionnaire was sent by email to key contacts from OH networks existing in the Region, only 13 countries answered the questionnaire, with at least one respondent per country. The Latin America region is made up of 21 countries and the Latin America and the Caribbean region is made up of 46 countries (47). The participation of at least two professionals from each of the three areas was expected in each Latin American country, since there is no contact in the Caribbean region. That is, we obtained approximately 60% of the expected response rate. The mode of distribution of the survey somewhat limited the number of responses, due to the fact that our study was an independent investigation. In this sense, Cole (48), in his comparative study between web surveys and surveys sent by mail, ensures that surveys that reach personal mail with their own name, are 39% more likely to be answered than those that are posted on the web or they are sent by a third party. According to the same author, web surveys or surveys sent by third parties have a possibility of approximately 16.6% of being opened by people, but without submitting any response. This was observed in our study given that three people started to respond but gave up at some point. In the case of our study, the vast majority of respondents to the survey were professionals known by one of the authors and to whom the survey was sent to their respective personal emails with its own name. The apparent low participation may be a consequence of the breakdown of the OH union in Latin America, since the people belonging to each key area have not been clearly identified. However, since the participation to this survey was completely voluntary, the lack of interest to join this survey can be an indicator of the barriers for the operationalization of OH initiatives in the Region.

Our study differed from the European one on the sampling method, due to the lack of a baseline database of professionals working within this OH approach in Latin America. The survey form was firstly distributed to professionals from the Sapuvet network and the professional connections of the authors working in the government (public health and animal health) or in other high education institutions knowing or applying the concept of OH in Colombia. This may have created a bias on the type respondents who participated in the study, as the respondents from Colombia were more than a half (55%). Indeed, a known systemic network of OH in the region was absent at the time of the circulation of the survey. Besides, authors did not have control over how the survey reached the government in each country if so.

However, the results were analyzed in two different ways, one including Colombia and another one omitting Colombia's answers; when comparing these groups of analysis, results were very similar. We consider that other countries that did not participate in this survey could have similar results. Indeed, the OH concept became increasingly known as the norm to work during the response to the 2009–2010 influenza pandemic at the global, regional, national, and community level (49), but progress on the adoption and implementation of this approach has been slow in the Americas region. However, it is possible that in countries such as Brazil and Mexico, with a history of stronger OH collaboration, the perception and knowledge in this field may be different, due to the presence of avian flu (H1N1) in Mexico in 2009 (50), and in Brazil due to the PAHO Office presence and influence. In a recent publication, Pettan-Brewer et al. (46), reported that OH Brazil network has been a successful example to all other countries of inclusive and sustainable interdisciplinary partnerships uniting a country with national and international collaborations through OH. The network has established mutual official partnerships with organizations such as One Health Platform, One Health Initiative, One Health Commission, One Health Sweden, continuing to build solid partnerships among uncountable international organizations from all continents (46).

Another limitation of our study was the low participation of environmental science professionals in this survey. Although an attempt was made to contact and to invite professionals from the environmental sector no response was obtained. This limitation is also described by the World Bank stating that while environment is one of three main sectors in the concept of OH, in practice it is systematically underrepresented. The chronic lack of economic, and even ecological data available on impacts to the environment sector was a recurring discussion point (15). Nonetheless, some authors affirm that among the problems to include and to gather ecosystem field in the Latin American countries are the institutional weakness (absent or precarious human, technical, and financial resources), resulting in absent or discontinuous ecosystem monitoring programs, with low quality of available data (29). Briggs et al. (51), also provided evidence of the limited documentation, coverage, and accessibility of information about environmental initiatives in Latin America. More recently, Vizeu-Pinheiro et al. (52), unveil that most countries in Latin America have environmental laws but there are gaps between the laws and implementation in practice, also they revealed a great variation across countries and dimensions of environmental governance. The Environmental Rule of Law Regulatory agencies face implementation challenges, driven in part by constraints on human and financial capacity. While the region shows progress on environmental impact assessments, progress is still needed toward producing comprehensive explanations of agency decisions. Within civic engagement, the region has made progress on access to information but public participation remains a challenge and the rights of environmental defenders are a huge concern (52).

In the particular case of Colombia, Agudelo et al. (53) mentioned that from 2001 to 2014, some laws and plans, regarding the environment and its connectedness with health were created, as for example, the Public Health Ten-Year plan (53). In this Plan, the dimension called Environmental Health includes programs toward the prevention and control of zoonoses, the water and sanitation quality, the air quality and impact of pollution, the control of vector-borne diseases and vector control, the solid waste management, the surveillance of environmental risk factors, among others. This is a great improvement regarding environmental health, but it is premature to say that the changes are evident at this time (53, 54), because in the rural settings of the country, long historic social gaps have been indicating the abandonment of the Colombian State with the rural populations, especially people belonging to indigenous and afro descendants' groups. Those populations have the worst indicators in health, in terms of the maternal mortality rates, access to clean water, to primary health services, to sanitation of waste management and good house quality (55). In this way, we agree with Garnier et al. (56), pointing out that integrating a gender perspective together with the vision, traditional knowledge, and needs of Indigenous Peoples and Local communities, into a multi-sectoral OH approach, would greatly enhance biodiversity conservation, global health, and sustainable development outcomes. An organized approach to build collaborations between practitioners, community, and academia under the gender perspective, could improve environmental integration, biodiversity conservation, and OH implementation in Latin America, as women have a pivotal role in managing and conserving natural resources in the current challenges that emerge at the Human-Animal Environment interface (56). We believe that in Latin America countries there are auspicious biological and cultural scenarios to integrate a framework of gender-responsive and right based OH Approach that could help reverse the environmental, health, and climate degradation and loss of biodiversity and doing this becoming an example of socio-ecological resilience.

Finally, authors consider these results reflect a perceived need for change from a fragmented health organization to an integrated health response to global challenges not only in Colombia but also in other Latin America countries. We emphasize the urgency to integrate a framework for OH governance. In this sense, the stages of policy development based on knowledge integration (KI) as a mechanism for multi-institutional learning to improve the governance, and coordination of OH implementation as described by Hitziger et al. (57) are recommended. Along the development of health policies, the KI can be used to build a common framework enabling an understanding of the links between the knowledge of multiple individuals. In practice KI is a multidimensional challenge because it requires the integration of cognitive concepts, organizational, and social interests and perspectives as well as communicative and cultural factors. As shown in our results (Figures 8, 10), respondents attributed low scores to the level of implementation and awareness of OH in citizens, echoing how insufficient and limited the approach is in for the participants. The integration of the three forms of knowledge throughout a policy cycle can be facilitated by three different approaches: multicriteria analyses for target knowledge, systems thinking for systems knowledge and transdisciplinary approaches for transformation knowledge (57).

In these particular times, health programs are targeting an integrative approach for COVID-19, considering the role of OH initiatives (46). The year 2020 was key, since governments around the globe reviewed their progress on the Sustainable Development Goals, the Paris Agreement and the Convention on Biological Diversity. What we are going through as a species confirms the importance of accepting a new global agreement between nature and people. The most important lessons learned from this health, social, and environmental crisis in Latin America are: (i) the need for more efficient and transparent management of resources that allows greater equity and access to health services, (ii) the importance of strengthening health education competencies at the community level, and (iii) the urgency to develop a greater degree of empathy toward all the species with whom we inhabit the planet, among others. The current framework of the human-animal-ecosystem interface in Colombia and some of the other Latin America countries is affected by fragmentation of health interests, programs, and sectors, a general lack of societal participation and by professional focus on very limited areas of expertise. In this way, we consider that integration and implementation of the OH approach can support countries to improve their health policies and health governance as well as to advocate the social, economic, and environmental sustainability of the Region.

## Data Availability Statement

The original contributions presented in the study are included in the article/Supplementary Material, further inquiries can be directed to the corresponding author/s.

## Ethics Statement

The studies involving human participants were reviewed and approved by Ethical approval was granted by the Clinical Research and Ethical Review Board at the Royal Veterinary College, grant holder of COST Action TD1404 NEOH (ref. prot. n. URN 2016 1554). The patients/participants provided their written informed consent to participate in this study.

## Author Contributions

The article is the result of a survey. NC: senior author supervising the writing, translation of the survey, general coordination and supervision of the questionnaire distribution, managing questionnaire answers, and drafting and editing manuscript. LT, FC, and DM: conceptualization and design of the questionnaire survey. AO: data cleaning and managing questionnaire answers, data management and analysis, snd creation of figures and tables. NC, AO, LT, FC, and DM: survey and article editing. All authors reviewing the final version of the manuscript.

## Conflict of Interest

The authors declare that the research was conducted in the absence of any commercial or financial relationships that could be construed as a potential conflict of interest. The handling editor is currently organizing a research topic with one of the authors NC.

## Publisher's Note

All claims expressed in this article are solely those of the authors and do not necessarily represent those of their affiliated organizations, or those of the publisher, the editors and the reviewers. Any product that may be evaluated in this article, or claim that may be made by its manufacturer, is not guaranteed or endorsed by the publisher.
