# Supplementary material for: A Survey on One Health Approach in Colombia and Some Latin American Countries: From a Fragmented Health Organization to an Integrated Health Response to Global Challenges
Source: Front Public Health. 2021 Oct 25;9:649240. doi: 10.3389/fpubh.2021.649240 (PMC8573084; doi:10.3389/fpubh.2021.649240)
Supplement: Supplementary file 1 [file Presentation_1.pdf]

ENCUESTA UNA SALUD

- ÁMBITO DEL CUESTIONARIO Y DETALLES -
Esta encuesta se ha promovido bajo la iniciativa de COST Actions TD1404 "Red de evaluación de One Health-NEOH".

El propósito de este cuestionario es recopilar información y datos sobre la existencia / implementación de acciones / iniciativas de One Health (OH) en su país. Además de recopilar información sobre iniciativas OH existentes o planificadas en países de América Latina.

Para tener una encuesta representativa, el cuestionario OH debe ser respondido por los encuestados clave que representan los tres componentes OH, Salud Animal, Salud Humana / Salud Pública y Salud Ambiental, representados por los siguientes subgrupos principales de encuestados:
• instituciones / ministerios públicos
• academia / investigación
• sector privado
• ONG y otras partes interesadas
Le recomendamos que solo responda las preguntas cubiertas por su experiencia.

La encuesta no debe tomar más de 10-15 minutos para completarse.

- DESCARGO DE RESPONSABILIDAD -
Al completar y enviar, acepto voluntariamente participar.
ii. Tus respuestas permanecerán en el anonimato Nadie podrá identificarlo ni a usted ni a sus respuestas, y nadie sabrá si participó o no en el estudio.
iii. La información recopilada puede no beneficiarlo directamente, pero la información que se aprende en este estudio debe proporcionar beneficios más generales para los fines de investigación del proyecto.
iv. Sus respuestas se enviarán a un enlace en https://docs.google.com donde los datos se almacenarán en un formato electrónico protegido con contraseña. docs.google.com no recopila información de identificación como su nombre, dirección de correo electrónico o dirección IP.

Por favor, tenga la amabilidad de completar el cuestionario a fines de agosto de 2018.
Gracias de antemano por su amable colaboración y apoyo.

-RECONOCIMIENTO-
Compatible con COST Action TD1404 (NEOH), con el soporte de COST (Cooperación Europea en Ciencia y Tecnología)

Para obtener más información acerca de NEOH, visite:
http://neoh.onehealthglobal.net/
http://www.cost.eu/COST_Actions/tdp/TD1404?parties

Para cualquier consulta relacionada con el cuestionario, puede ponerse en contacto con Natalia Cediel (nmcedielb@unisalle.edu.co), Daniele De Meneghi (daniele.demeneghi@unito.it), Francesco Chiesa (francesco.chiesa@unito.it).

1. INFORMACIÓN GENERAL

Pregunta 1.1

¿Cuál es el país donde usted trabaja? *

Elige

PREGUNTA 1.2

Cual es su disciplina? *

Ciencias Ambientales
Salud Humana
Ciencias Animales
Salud pública
Otro:

Por favor, especifique su título universitario

Tu respuesta

PREGUNTA 1.3

En qué tipo de institución está empleado? *

Elige

*

Disciplina a la que pertenece la institución

Salud Ambiental
Ciencias animales
Salud Humana
Salud Pública
Otro:

Cuál es su posición actual en la institución?

2. SOBRE "ONE HEALTH"

PREGUNTA 2.1

Es usted miembro de algún proyecto o red sobre One Health? *

Si
No

En caso afirmativo "Si", por favor, especifique

Tu respuesta

Si no, ha escuchado hablar de OH?

Si
No

PREGUNTA 2.2

Qué entiende por Una Salud "One Health"?
Por favor, defina OH en una frase

Tu respuesta

PREGUNTA 2.3

Está actualmente involucrado en iniciativas de OH?(e.j. vigilancia, prevención y control de: zoonosis transmitidas por vectores, contaminantes ambientales en los alimentos, rabia, etc.)

Si
No

En caso afirmativo, por favor dé una breve descripción de las iniciativas

Tu respuesta

PREGUNTA 2.4

Su Institución ha respaldado/adoptado OH?

Si
No
Sin respuesta/No sabe

En caso afirmativo, responda cómo

Tu respuesta

PREGUNTA 2.5

Por favor liste programas / actividades en las cuales su institución sigue un enfoque OH, si aplica

Tu respuesta

PREGUNTA 2.6

Qué tan relevantes son las siguientes ventajas de One Health descritas en la literatura?

Califique desde 1 (no relevante) hasta 5 (altamente relevante)

1
2
3
4
5

- Detección temprana de amenazas y respuesta rápida, oportuna o efectiva
- Control de enfermedades y medidas de bioseguridad más efectivas
- Beneficio económico incremento en la eficiencia económica
- Mejoramiento del la salud y bienestar humano y animal
- Mayor calidad o mayor cantidad de información / datos y habilidades y conocimiento mejorados
- Beneficio al ecosistema
- Beneficios personales o sociales
- Diseño de políticas en salud
- Detección temprana de amenazas y respuesta rápida, oportuna o efectiva
- Control de enfermedades y medidas de bioseguridad más efectivas
- Beneficio económico incremento en la eficiencia económica
- Mejoramiento del la salud y bienestar humano y animal
- Mayor calidad o mayor cantidad de información / datos y habilidades y conocimiento mejorados
- Beneficio al ecosistema
- Beneficios personales o sociales

- Diseño de políticas en salud

Por favor, liste cualquier ventaja adicional:

Tu respuesta

PREGUNTA 2.7

Existen asociaciones/comités/consejos activamente tratando los problemas o iniciativas en OH en su país?

Si
No
Sin respuesta/ No sé

En caso afirmativo, por favor proporcione o detalle la iniciativa, participantes y problemáticas discutidas

Tu respuesta

PREGUNTA 2.8

Conoce si hay conexiones FORMALES entre la administración en salud pública y salud animal/veterinaria en su país (instituciones o servicios gubernamentales) ?

Si
No

En caso afirmativo, por favor explique la autoridad, nivel, responsabilidades y título del cuerpo existente

Tu respuesta

A qué nivel?

Nacional
Sub-nacional (i.j. regional, provincial)
Local

Naturaleza de la cooperación

Intercambio de información / datos
Vigilancia conjunta
Presupuesto compartido
intervenciones conjuntas

Preparación conjunta
Entrenamiento conjunto
Políticas de salud
Otro:

Cooperación formal (si hay) establecida en

últimos 5 años
últimos 5 a 10 años
últimos 10 a 20 años
últimos 20 a 30 años
Más de 30 años

PREGUNTA 2.9

En su conocimiento y opinión personal, existe alguna iniciativa de OH que esté siendo implementada en su país?

Si
No
Sin respuesta/ no sé

En caso afirmativo, por favor indique cuántas

1-5
6-10
>10

Por favor marque los campos de actividades en donde estas iniciativas son implementadas:

Vigilancia y monitoreo de enfermedades
Prevención y control de enfermedades
Conocimiento por parte de los participantes de los programas
Programas de educación superior
Investigación
Otro:

Cuáles son los tópicos específicos cubiertos por estas iniciativas? Por favor proporcione la lista (preferiblemente con enlaces web) de iniciativas OH que usted refiere

Tu respuesta

En caso negativo, por favor detalle los campos de actividades en donde usted piensa que estos programas deberían ser implementados

Vigilancia y monitoreo de enfermedades
Prevención y control de enfermedades

Conocimiento por parte de los participantes de los programas
Programas de educación superior
Investigación
Otro:

PREGUNTA 2.10

En su conocimiento, cuales de los siguientes profesionales están directamente empleados/involucrados en iniciativas de OH en su país?

Veterinarios
Médicos
Pediatras
Médicos familiares
Biológos/entomólogos
Químicos
Environmental/ecosystem specialists (ecologists)
Sociology/anthropology/gender specialists
No answer/I don't know
Otro:

3. ENFERMEDADES ZOONÓTICAS, SALUD AMBIENTAL Y RESISTENCIA ANTIMICROBIANA: EJEMPLOS DE PROBLEMAS (URGENTES) EN ONE HEALTH

PREGUNTA 3.1

Existe una cooperación activa entre el ministerio de salud y el ministerio responsable de medicina veterinaria cuando se ocupan de las zoonosis?

Si
No
Sin respuesta/No sé
Otro:

En caso afirmativo, dé un ejemplo

Tu respuesta

PREGUNTA 3.2

Existe una norma legislativa o regla que regule la colaboración entre los servicios de Salud Pública y Veterinarios (obligación de garantizar un flujo reciproco de información / datos)?

Si
No
Sin respuesta/No sé

En caso afirmativo, describa brevemente

Tu respuesta

PREGUNTA 3.3

Seleccione entre las enfermedades zoonóticas listadas cuales están controladas y monitoreadas por el Ministerio de Salud y/o de Agricultura
Lista basada en: Zoonosis y la Interface Humano-Animal-Ecosistemas
(http://www.who.int/zoonoses/en/)

Ministerio de Salud
Ministerio de Agricultura
Ambos

- Anthrax
- Influenza Aviar
- Brucelosis
- Campylobacter
- Enfermedad de Chagas
- Chikungunya
- Cisticercosis
- Dengue
- Virus de Ebola, Fiebre Hemorrágica de Crimea-Congo, Lassa y Marburg
- Leptospirosis
- Peste (Yersinia pestis)
- Fiebre Q
- Rabia
- Fiebre del Valle de Rift
- Dermatofitosis (Tiña)
- Salmonella
- Toxoplasmosis
- Triquinelosis
- Tularemia
- Encefalitis Equina Venezolana
- Virus del Nilo Occidental
- Virus Zika

- Anthrax
- Influenza Aviar
- Brucelosis
- Campylobacter
- Enfermedad de Chagas
- Chikungunya
- Cisticercosis
- Dengue
- Virus de Ebola, Fiebre Hemorrágica de Crimea-Congo, Lassa y Marburg
- Leptospirosis
- Peste (Yersinia pestis)
- Fiebre Q
- Rabia
- Fiebre del Valle de Rift
- Dermatofitosis (Tiña)
- Salmonella
- Toxoplasmosis
- Triquinelosis
- Tularemia
- Encefalitis Equina Venezolana
- Virus del Nilo Occidental
- Virus Zika

PREGUNTA 3.4

Cuál es el nivel de conocimiento por parte de la comunidad sobre enfermedades de animales expuestos a contaminantes ambientales y posteriormente transmitidas a humanos por alimentos de origen animal (Ejemplo: dioxinas, PCB)?

pobre/sin conocimiento

1
2
3
4
5

conocimiento excelente

No competente en este campo

PREGUNTA 3.5

Cuál es la calidad de los planes nacionales para la prevención y el monitoreo de las enfermedades transmitidas por alimentos de origen animal originadas por contaminantes ambientales?

pobre

1
2
3
4
5

excelente

No competente en este campo

PREGUNTA 3.6

Su país contribuye al monitoreo global de RAM (resistencia antimicrobiana) con un programa específico de monitoreo e investigación?

Si
No
Sin respuesta/No sé

En caso afirmativo, describa por favor

Tu respuesta

4. ASPECTOS LIMITANTES DE LA INTERDISCIPLINARIDAD/INTERSECTORIALIDAD

PREGUNTA 4.1

Si usted ha trabajado en OH en su país, cuáles han sido los factores limitantes la interdisciplinariedad/intersectorialidad?

Tu respuesta

PREGUNTA 4.2

Describa el nivel y las oportunidades para la colaboración en su país dentro de:

N/A
Pobre
Suficiente
Bueno
Excelente

Consejos Profesionales (con especial atención a aquellos veterinarios y médicos)
Cursos universitarios
Instituciones (ministerios y/o servicios) reguladores que manejan la vigilancia veterinaria y el control de la cadena alimentaria
Instituciones (ministerios y/o servicios) responsables de las emergencias, incluidos el manejo de desastres, la rehabilitación y resiliencia

5. CONCLUSIONES

PREGUNTA 5.1

Evalúe qué tan bien el enfoque OH es implementado por los profesionales empleados/involucrados en los sectores de salud veterinaria, pública y ambiental en su país

pobre
1
2
3
4
5
excelente

Sin respuesta / No sé

PREGUNTA 5.2

Existen iniciativas recientes (formales) para establecer/fortalecer la colaboración intersectorial (a nivel administrativo/académico) que busquen apoyo global al enfoque OH?

Si
No
Sin respuesta/No sé
Otro:

En caso afirmativo, por favor describa brevemente

Tu respuesta

PREGUNTA 5.3

Cuáles son los tres problemas mas importantes en salud ambiental, animal y humana en su país en los últimos 5 años?

Tu respuesta

PREGUNTA 5.4

Por favor liste mínimo 3 instituciones las cuales coordinan o son responsables por actividades de One Health en su país

Tu respuesta

Sin respuesta/No sé

PREGUNTA 5.5

En su opinión dónde y cuáles son las brechas/vacíos de los planes en One Health?

Tu respuesta

Sin respuesta/No sé

PREGUNTA 5.6

En su opinión, cuál es el nivel de conocimiento/percepción de OH entre los ciudadanos/consumidores de su país?

Ninguna

1
2
3
4
5

Excelente

Cuáles iniciativas podrían ser emprendidas para desarrollar/mejorar tal conocimiento?

Tu respuesta

6. Fin de la Encuesta

Comentarios u observaciones que quisiera añadir

Tu respuesta

Sugerencias para mejorar la encuesta

Tu respuesta

GRACIAS POR COMPLETAR NUESTRA ENCUESTA
